# Allylic Alkylations Catalyzed By Palladium-Bis(oxazoline) Complexes Derived From Heteroarylidene Malonate Derivatives

**DOI:** 10.3390/molecules17021992

**Published:** 2012-02-17

**Authors:** Lei Liu, Hongli Ma, Bin Fu

**Affiliations:** Department of Applied Chemistry, China Agricultural University, Beijing 100193, China; Email: LiuLei-111@163.com (L.L.); hongli08@126.com (H.M.)

**Keywords:** bis(oxazoline), asymmetric catalysis, allylic alkylation

## Abstract

A series of simple heteroarylidene malonate-type bis(oxazoline) ligands **4** and **5** were applied to the Palladium-catalyzed allylic alkylation reaction, and the ligand **4a** bearing a phenyl group afforded excellent enantioselectivity (up to 96% *ee*) for the allylic alkylation product. Other substrates were also examined, giving the allylic alkylated products in high yield but with poor *ee* values.

## 1. Introduction

Palladium-catalyzed asymmetric allylic substitution is a versatile, widely used process in organic synthesis for the enantioselective formation of C–C and C-heteroatom bonds. A number of chiral ligands with P, N, and S as coordinating atoms have been synthesized and applied to this transformation [1,2,3,4,5,6]. Most of the efficient chiral ligands developed for this reaction are mixed bidentate donor ligands with P–N chelating mode [5,7]. In general, examples of *N*,*N*-chelating ligands for catalytic allylic alkylation are also abundant although they are less common than other types of ligands. During the past two decades, chiral bis(oxazolines) (BOX), as a class of typical *N*,*N*-bidentate ligand, are among the most widely studied ligands in catalytic asymmetric synthesis. As a result, some bis(oxazolines) have been also applied to the palladium-catalyzed allylic alkylation and demonstrated high enantioselectivity [8,9,10,11,12,13,14,15].

**Figure 1:**
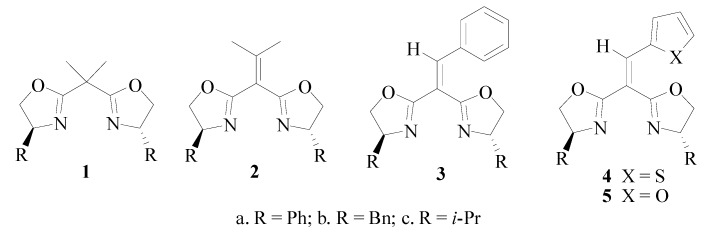
The alkylidene and arylidene malonate-type bis(oxazoline) ligands.

Among the bis(oxazoline) ligands with diverse skeletons and backbones reported, malonate-type BOX **1** is one of the most representative classes. In this type of ligands, the bridge angle, correlating with the bite angle of BOX-metal complex, is considered as an important structural factor influencing the enantioselectivity [16,17,18]. In recent years, some alkylidene or arylidene malonate-type BOX ligands such as **2**, **3**, **4** and **5** (Figure 1) [19,20,21,22], have been reported to demonstrate good enantioselectivity in different asymmetric catalytic reactions. Especially, our group found that ligands **4** and **5** with furan or thiophene units displayed excellent asymmetric catalytic properties in the Cu(II) catalyzed Friedel-Crafts alkylation of indole derivatives with arylidene malonates (99% yield and up to >99% *ee*) [22]. As seen from the previous studies, both the substituent on the oxazoline ring and the heterocycle moiety attached to the other end of the double bond play important roles in the asymmetric catalysis owing to their different steric and electronic effects. In our ongoing efforts to explore highly enantioselective reactions using simple and cheap chiral catalytic systems, the heteroarylidene malonate-type bis(oxazolines) **4** and **5** were further applied in the allylic alkylation reaction. Herein, we report our recent progress on this subject.

## 2. Results and Discussion

The allylic alkylation, as a powerful carbon-carbon bond formation, has attracted the attention of many chemists [23,24,25,26,27,28,29]. In order to expand the potential of the ligands **4** and **5** with thiophene or furan units, we applied them in the palladium-catalyzed asymmetric allylic alkylation of 1,3-diphenyl-2-propen-1-yl acetate with dimethyl malonate, which is commonly used as a model reaction for evaluation of chiral catalysts in much of the literature. The reaction was catalyzed by 5 mol% complexes generated *in situ* from 2.5 mol % of [Pd(η^3^-C_3_H_5_)Cl]_2_, 6 mol % of the chiral ligands, in the presence of *N*,*O*-bis(trimethylsilyl)acetamide (BSA) and 20 mol % of KOAc in dichloromethane. As summarized in Table 1, the reaction worked well at room temperature to give the products in high yields within 24 h (entries 1–6). High asymmetric induction was observed for most of the ligands tested. Four ligands, namely **4a**, **4b**, **5a** and **5b**, showed almost the same high enantioselectivity, and the best 92% *ee* was obtained by thienylidene BOX **4a** with a phenyl substituent on the oxazoline ring. The enantioselectivity can be further improved to 96% at 0 °C, although the reaction time was prolonged to 48 h (entry 7). Reactions in other solvents only gave inferior yields and enantioselectivities (entries 8–10). 

**Scheme 1:**
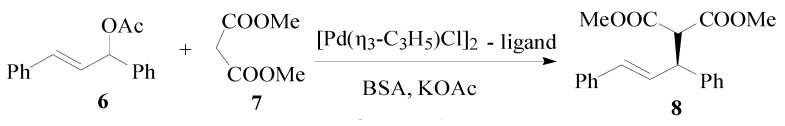
The model reaction of palladium-catalyzed asymmetric allylic alkylation.

**Table 1:** Effect of ligands and solvent in the Pd-catalyzed allylic alkylation ^a^.

Entry	Ligands	Solvent	Temp. (°C)	Time (h)	Yield ^b^ (%)	*ee* ^c^ (%)
1	**4a**	DCM	20	24	85	92
2	**4b**	DCM	20	24	85	90
3	**4c**	DCM	20	24	80	47
4	**5a**	DCM	20	24	90	91
5	**5b**	DCM	20	24	86	89
6	**5c**	DCM	20	24	80	15
7	**4a**	DCM	0	48	80	96
8	**4a**	ClCH_2_CH_2_Cl	0	48	85	84
9	**4a**	Toluene	0	48	70	76
10	**4a**	CH_3_CN	0	48	65	52

^a^ All the reactions were conducted under nitrogen using 5 mol % of catalyst; ^b^ Isolated yield; ^c^ Determined by chiral HPLC.

Encouraged by the excellent results, we next investigated the allylic alkylation of other substrates by varying different acetate esters, as illustrated in Table 2. When 1-methyl-3-phenyl-2-propen-1-yl acetate (**9**) reacted with dimethyl malonate under the same catalytic conditions, the allylic product was obtained in high yield, but with a very poor ee value (5%, entry 1). Subsequently, the substrates 1-phenyl-2-propen-1-yl acetate (**11**) and 1-naphthyl-2-propen-1-yl acetate (**14**) were tested, but to our disappointment, none of the desired allylic alkylation products **12** and **15** were obtained, and rather the linear products **13** and **16** without any chiral element were produced in high yield (entries 2 and 3). This phenomenon is basically in agreement with that most of the Pd catalysts developed to date favor the formation of the achiral linear product rather than the desired branched isomer [30,31]. Furthermore, the reaction of cyclic acetates **17** and **19** with dimethyl malonate were tested. Racemic products **18** and **20** were obtained in high yield, respectively (entries 4 and 5). In addition, we examined the reaction of methyl 1-tetralone-2-carboxylate with cinnamyl acetate or allyl acetate, respectively [32]. Unfortunately, despite the high yield the allylic alkylated products, these were obtained in racemic form.

**Table 2:** Palladium-4a complex catalyzed allylic alkylation of various substrates^a^.

Entry	Substrates		Desired product ^a^	Actual product ^b^	*ee* ^c^
**1**	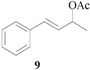	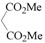		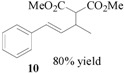	5%
**2**	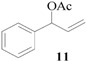	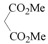	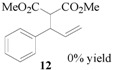	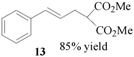	0
**3**	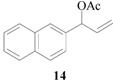	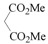	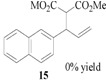	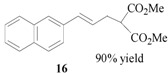	0
**4**	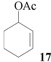	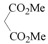		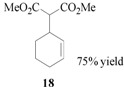	0
**5**	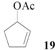	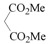		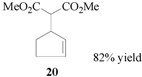	0
**6**	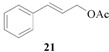	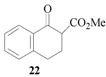		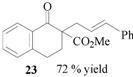	0
**7**	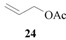	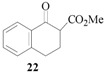		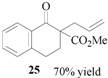	0

^a^ All the reactions were conducted under nitrogen using 5 mol % of catalyst; ^b^ Isolated yield; ^c^ Determined by chiral HPLC.

## 3. Experimental

### 3.1. General

NMR spectra were recorded with a Bruker Avance DPX300 spectrometer with tetramethylsilane as the internal standard. Infrared spectra were obtained on a Nicolet AVATAR 330 FT-IR spectrometer. Optical rotations were measured on a Perkin-Elmer 341 LC polarimeter. The enantiomeric excesses of (*R*)- and (*S*)-enantiomer were determined by HPLC analysis over a chiral column (Daicel Chiralcel AD-H or OD-H; eluted with hexane-isopropyl alcohol; UV detector, 254 nm). The absolute configuration of the major enantiomer was assigned by comparison with literature. Solvents were purified and dried by standard procedures.

### 3.2. General Procedure for Catalytic Asymmetric Allylic Alkylation

#### *(*S*-Dimethyl 2-[(*E*)-1,3-Diphenylprop-2-en-1-yl]malonate*) (**8**)

To a solution of ligand **4a** (12.0 mg, 0.03 mmol) in CH_2_Cl_2_ (1.0 mL) was added [Pd(η^3^-C_3_H_5_)Cl]_2_ (4.6 mg, 0.0125 mmol) and anhydrous KOAc (10 mg, 0.10 mmol). The resulting solution was stirred for 30 min, and 1, 3-diphenyl-3-acetoxy-1-propene (126 mg, 0.50 mmol) was then added as a solution in CH_2_Cl_2_ (1.0 mL), followed by dimethyl malonate (0.17mL, 1.5 mmol) and BSA (0.37 mL, 1.5 mmol). After stirring for 24 ~ 48 h at room temperature or 0 °C, the solution was quenched by the addition of saturated ammonium chloride solution (10 mL). The aqueous phase was extracted with EtOAc (2 × 10 mL), the combined organics were dried over anhydrous Na_2_SO_4_, and the solvent was removed *in vacuo* to yield an orange oil. Purification by chromatography on silica eluting with 20% EtOAc/hexane gave the product as a colorless oil. ^1^H-NMR (CDCl_3_): *δ *7.35–7.17 (m, 10H, ArH), 6.47 (d, *J* = 15.75 Hz, 1H, –CH=), 6.32 (dd, *J* = 8.43 Hz, 15.90 Hz, 1H, CH=), 4.26 (dd, *J* = 8.43 Hz, 10.80 Hz, 1H, CH), 3.95 (d, *J* = 10.80 Hz, 1H, CH), 3.71 (s, 3H, CH_3_O), 3.52 (s, 3H, CH_3_O). HPLC analysis (Chiralcel AD-H, *n*-hexane/*iso*-PrOH, 90:10, 1.0 mL/min, 254 nm): t_r_(minor) = 12.72 min, t_r_(major) = 18.54 min; 96% *ee*.

### 3.3. Dimethyl 2-[(*E*)-1-methyl-3-phenylprop-2-en-1-yl]malonate (**10**)

This compound was prepared according to the general procedure using *(E)-*1-methyl-3-phenylprop-2-en-1-yl acetate (**9**, 0.95 g, 0.50 mmol). The desired product **10** was obtained as a colorless oil. ^1^H-NMR (CDCl_3_): *δ* 7.35–7.20 (m, 5H, ArH), 6.45 (d, *J* = 15.70 Hz, 1H, –CH=), 6.12 (dd, *J* = 8.43 Hz, 15.90 Hz, 1H, CH=), 3.75 (s, 3H, CH_3_), 3.67 (s, 3H, CH_3_), 3.40 (d, *J* = 9.0 Hz, 1H, CH), 3.12 (m, 1H, CH), 1.19 (d, *J* = 6.60 Hz, 3H, CH_3_).

### 3.4. Dimethyl 2-[(*E*)-1-phenylprop-2-en-1-yl]malonate (**13**)

Prepared from *(E)-*1-phenylprop-2-en-1-yl acetate (**11**, 0.88 g, 0.50 mmol). The product **13** was obtained as a colorless oil. ^1^H-NMR (CDCl_3_): *δ* 7.34–719 (m, 5H, ArH), 6.47 (d, *J* = 15.80 Hz, 1H, –CH=), 6.18–6.09 (m, 1H, CH=), 3.75 (s, 6H, CH), 3.52 (q, 1H, *J* = 7.53 Hz, CH), 2.83–2.78(m, 2H, CH_2_).

### 3.5. Dimethyl 2-[(*E*)-3-naphthylprop-2-en-1-yl]malonate (**16**)

Prepared from *(E)*-1-naphthylprop-2-en-1-yl acetate (**14**, 1.13 g, 0.50 mmol). The product **16** was obtained as a colorless oil. ^1^H-NMR (CDCl_3_): *δ* 8.06 (t, *J* = 2.10 Hz, 1H, ArH), 7.83 (dd, *J* = 3.21 Hz, 6.60 Hz), 7.76 (d, *J* = 8.10 Hz, 1H, ArH), 7.54–739.(m, 5H, ArH), 7.25 (s, 1H, ArH), 7.20 (s, 1H, -CH=), 6.20–6.10 (m, 1H, CH=), 3.77 (s, 6H, 2 × CH_3_), 3.65–3.57 (dd, *J* = 7.53 Hz, 15.30 Hz, 1H, CH), 2.96–2.90(m, 2H, CH_2_).

### 3.6. Dimethyl 2-[cyclohex-2-en-1-yl]malonate (**18**)

Prepared from cyclohex-2-en-1-yl acetate (**17**, 0.70 g, 0.50 mmol). The product **18** was obtained as a colorless oil. ^1^H-NMR (CDCl_3_): *δ* 5.81–5.75 (m, 1H, =CH), 5.54–5.50 (m, 1H, CH=), 3.74 (s, 6H, 2 × CH_3_), 3.29 (d, *J* = 9.60 Hz, 1H, CH), 2.95–2.87 (m, 1H, –C*H*–CH=), 2.00 (m, 2H, C*H*_2_–CH=), 1.80–1.69 (m, 2H, CH_2_), 1.60–1.54 (m, 1H, one of CH_2_), 1,42–1.35(m, 1H, one of CH_2_).

### 3.7. Dimethyl 2-[cyclopent-2-en-1-yl]malonate (**20**)

From cyclopent-2-en-1-yl acetate (**19**, 0.63 g, 0.50 mmol). The product **20** was obtained as a colorless oil. ^1^H-NMR (CDCl_3_): *δ* 5.82–5.78 (m, 1H, =CH), 5.64–5.60 (m, 1H, CH=), 3.71(s, 6H, 2 × CH_3_), 3.36–3.30 (m, 1H, CH), 3.24(d, *J* = 9.60 Hz, 1H, CH), 2.35–2.26 (m, 2H, CH_2_), 2.15–2.05 (m, 1H), 1.62–1.52 (m, 1H, CH).

### 3.8. Methyl 2-carboxylate-2-cinnamyl 1-tetralone (**23**)

From *(E)-*cinnamyl acetate (**21**, 0.88 g, 0.50 mmol) and methyl 1-tetralone-2-carboxylate (**22**, 0.51 g, 0.25 mmol). The desired product **23** was obtained as a colorless oil. ^1^H-NMR (CDCl_3_): *δ* 8.07 (d, *J* = 1.23 Hz, 7.51 Hz, 1H, ArH), 7.50–7.45 (m, 1H, ArH), 7.35–7.17 (m, 7H, ArH), 6.48 (d, *J* = 15.69 Hz, 1H, CH=), 6.28–6.17 (m, 1H, CH=), 3.68 (s, 1H, OMe), 3.67 (s, 1H, CH), 3.40 (d, *J* = 9.0 Hz, 1H, CH), 3.12 (m, 1H, CH), 1.19 (d, *J* = 6.60 Hz, 3H, CH_3_).

### 3.9. Methyl 2-carboxylate-2-allyl 1-tetralone (**25**)

From *(E)-*allyl acetate (**24**, 0.50 g, 0.50 mmol) and methyl 1-tetralone-2-carboxylate (**22**, 0.51 g, 0.25 mmol). The desired product **25** was obtained as a colorless oil. ^1^H-NMR (CDCl_3_): *δ *8.06 (dd, *J* = 1.20 Hz, 7.65 Hz, 1H, ArH), 7.50–7.45 (m, 1H, ArH), 7.33–7.29 (m, 1H, ArH), 7.22 (d, *J* = 7.50 Hz, 1H, ArH), 5.87–5.75 (m, 1H, CH=), 5.18–5.08 (m 2H, CH_2_=), 3.67 (s, 1H, OMe), 3.07–2.93 (m, 2H, CH_2_), 2.72 (m, 2H, CH_2_), 2.55–2.49 (m, 1H, CH), .2.19–2.09 (m, 1H, CH).

## 4. Conclusions

In conclusion, a series of heteroarylidene malonate-type chiral bis(oxazoline) ligands **4** and **5** were applied to the palladium-catalyzed allylic alkylation. Ligand **4a** bearing a phenyl group afforded the highest ee (96%), which is comparable to the results obtained by previously reported BOX ligands [8,9,10,11,12,13,14,15]. Other substrates were also investigated and provided the allylic alkylated products in high yields but with poor enantioselectivities. The results indicate that our heteroarylidene malonate-type BOX ligands show promising potential application in asymmetric catalysis. Further studies to extend this catalytic system to other asymmetric reactions is now in progress in our laboratory.

## Supplementary Materials

Supplementary materials can be accessed at: http://www.mdpi.com/1420-3049/17/2/1992/s1.

## References

[1] Trost B.M., van Vranken D.L. (1996). Asymmetric transition metal-catalyzed allylic alkylations. Chem. Rev..

[2] Johannsen M., Jørgensen K.A. (1998). Allylic amination. Chem. Rev..

[3] Helmchen G., Pfaltz A. (2000). Phosphinooxazolines—A new class of versatile, modular P,N-ligands for asymmetric catalysis. Accounts Chem. Res..

[4] Trost B.M., Crawley M.L. (2003). Asymmetric transition-metal-catalyzed allylic alkylations:  Applicationsin total synthesis. Chem. Rev..

[5] Lu Z., Ma S. (2008). Metal-catalyzed enantioselective allylation in asymmetric synthesis. Angew.Chem. Int. Ed..

[6] Hartwig J.F., Stanley L.M. (2010). Mechanistically driven development of iridium catalysts for asymmetric allylic substitution. Accounts Chem. Res..

[7] Pàmìes O., Dìéguez M., Claver C. (2005). New phosphite-oxazoline ligands for efficient Pd-catalyzed substitution reactions. J. Am. Chem. Soc..

[8] Du X., Liu H., Du D.M. (2010). Rational tuning of the rigidity of a ligand scaffold: synthesis of diphenylsulfide-linked bis(oxazoline) ligands and their application in asymmetric allylic alkylation. Tetrahedron: Asymmetry.

[9] Aït-Haddou H., Hoarau O., Cramailére D., Pezet F., Daran J.-C., Balavoine G.G.A. (2004). New dihydroxy bis(oxazoline) ligands for the palladium-catalyzed asymmetric allylic alkylation: Experimental investigations of the origin of the reversal of the enantioselectivity. Chem. Eur. J..

[10] Bayardon J., Sinou D., Guala M., Desimoni G. (2004). Applications of enantiopure 4,5-diphenyl substituted box and pybox ligands in asymmetric catalysis. Tetrahedron: Asymmetry.

[11] Bayardon J., Sinou D. (2004). Enantiopure fluorous bis(oxazolines): Synthesis and applications in catalytic asymmetric reactions. J. Org. Chem..

[12] Pericás M.A., Puigjaner C., Riera A., Vidal-Ferran A., Gómez M., Jiménez F., Muller G., Rocamora M. (2002). Modular bis(oxazoline) ligands for palladium catalyzed allylic alkylation: Unprecedented conformational behaviour of a bis(oxazoline) palladium η3-1,3-diphenylallyl complex. Chem. Eur. J..

[13] Glos M., Reiser O. (2000). Aza-bis(oxazolines): New chiral ligands for asymmetric catalysis. Org. Lett..

[14] Mcmanus H.A., Guiry P.J. (2004). Recent developments in the application of oxazoline-containing ligands in asymmetric catalysis. Chem. Rev..

[15] Hargaden G.C., Guiry P.J. (2009). Recent applications of oxazoline-containing ligands in asymmetric catalysis. Chem. Rev..

[16] Davies I.W., Gerena L., Castonguay L., Senanayake C.H., Larsen R.D., Verhoeven T.R., Reider P.J. (1996). The influence of ligand bite angle on the enantioselectivity of copper(II)-catalysed Diels-Alder reactions. Chem. Commun..

[17] Davies I.W., Deeth R.J., Larsen R.D., Reider P.J. (1999). A CLFSE/MM study on the role of ligand bite-angle in Cu(ΙΙ)-catalyzed Diels-Alder reaction. Tetrahedron Lett..

[18] Denmark S.E., Stiff C.M. (2000). Effect of ligand structure in the bisoxazoline mediated asymmetric addition of methyllithium to imines. J. Org. Chem..

[19] Carreiro E.P., Chercheja S., Burke A.J., Ramalho J.P., Rodrigues A.P. (2005). Isbut-Box: A new chiral C_2_ symmetric bis-oxazoline for catalytic enantioselective synthesis. J. Mol. Catal. A Chem..

[20] Carreiro E.P., Chercheja S., Moura N., Gertrudes S.C., Burke A.J. (2006). Arylid-Box: A new family of chiral bis-oxazoline ligands for metal mediated catalytic enantioselective synthesis. Inorg. Chem. Commun..

[21] Burke A.J., Carreiro E.P., Chercheja S., Moura N.M.M., Ramalho J.P., Rorigues A.I., Santos C.I.M. (2007). Cu(I) catalysed cyclopropanation of olefins: Stereoselectivity studies with Arylid-Box and Isbut-Box ligands. J. Organomet. Chem..

[22] Sun Y.J., Li N., Zheng Z.B., Liu L., Yu Y.B., Qin Z.H., Fu B. (2009). Highly enantioselective Friedel-Crafts reaction of indole with alkylidenemalonates catalyzed by heteroarylidene malonate-derived bis(oxazoline) copper(II) complexes. Adv. Synth. Catal..

[23] Kultyshev R.G., Miyazawa A. (2011). Ugi amine-derived P,N- and P,P-ligands with *N*-alkyltriethoxysilyl tethers: Synthesis and evaluation of mesoporous silica-supported Pd complexes in asymmetric allylic substitution reactions. Tetrahedron.

[24] Dugal-Tessier J., Dake G.R., Gates D.P. (2010). Verticilactam, a new macrolactam isolated from a microbial metabolite fraction library. Org. Lett..

[25] Thiesen K.E., Maitra K., Olmstead M.M., Attar S. (2010). Synthesis and characterization of new, chiral P–N ligands and their use in asymmetric allylic alkylation. Organometalics.

[26] Cattoën X., Pericás M.A. (2009). Synthesis of highly modular bis(oxazoline) ligands by Suzuki cross-coupling and evaluation as catalytic ligands. Tetrahedron.

[27] Tian F.T., Yao D.M., Zhang Y.J., Zhang W.B. (2009). Phosphine-oxazoline ligands with an axial-unfixed biphenyl backbone: The effects of the substituent at oxazoline ring and P phenyl ring on Pd-catalyzed asymmetric allylic alkylation. Tetrahedron.

[28] Diéguez M., Pámies O. (2008). Modular phosphite-oxazoline/oxazine ligand library for asymmetric Pd-catalyzed allylic substitution reactions: Scope and limitations-origin of enantioselectivity. Chem. Eur. J..

[29] Vargas F., Sehnem J.A., Galetto F.Z., Braga A.L. (2008). Modular chiral β-selenium-, sulfur-, and tellurium amides: Synthesis and application in the palladium-catalyzed asymmetric allylic alkylation. Tetrahedron.

[30] Hilgraf R., Pfaltz A. (1999). Chiral bis(*N*-tosylamino)phosphine- and TADDOL-phosphite-oxazolines as ligands in asymmetric catalysis. Synlett.

[31] You S.L., Zhu X.Z., Luo Y.M., Hou X.L., Dai L.X. (2001). Highly regio- and enantioselective Pd-catalyzed allylic alkylation and amination of monosubstituted allylic acetates with novel ferrocene P,N-ligands. J. Am. Chem. Soc..

[32] Trost B.M., Schroeder G.M. (1999). Palladium-catalyzed asymmetric alkylation of ketone enolates. J. Am. Chem. Soc..

