# Supplementary material for: Allylic Alkylations Catalyzed By Palladium-Bis(oxazoline) Complexes Derived From Heteroarylidene Malonate Derivatives
Source: Molecules. 2012 Feb 17;17(2):1992–9. doi: 10.3390/molecules17021992 (PMC6268680; doi:10.3390/molecules17021992)
Supplement: Supplementary file 1 [file molecules-17-01992-s001.doc]

**Supplementary Materials**

**The Allylic Alkylation Catalyzed By Palladium-Bis(oxazoline) Complex Derived From Heteroarylidene Malonate Derivatives**

**HPLC Diagram for Allylic Alkylation**

Reaction catalyzed by [Pd(η^3^-C_3_H_5_)Cl]_2_–**4a** in CH_2_Cl_2_ at room temperature.

# Time Area Height Width Area% Symmetry

1 12.284 4167.4 203.7 0.3028 3.946 0.467

2 16.894 101433.7 2584.8 0.6253 96.054 0.352

Reaction catalyzed by [Pd(η^3^-C_3_H_5_)Cl]_2_–**4b** in CH_2_Cl_2_ at room temperature.

# Time Area Height Width Area% Symmetry

1 11.756 4520 255.1 0.2693 5.174 0.613

2 15.685 82842.7 2573.8 0.5124 94.826 0.435

Reaction catalyzed by [Pd(η^3^-C_3_H_5_)Cl]_2_–**4c** in CH_2_Cl_2_ at room temperature.

# Time Area Height Width Area% Symmetry

1 12.407 9052.8 386.1 0.3908 26.819 0.457

2 18.65 24702.3 767.9 0.4932 73.181 0.612

Reaction catalyzed by [Pd(η^3^-C_3_H_5_)Cl]_2_–**5a** in CH_2_Cl_2_ at room temperature.

# Time Area Height Width Area% Symmetry

1 11.756 2693.7 152.8 0.2683 4.456 0.657

2 15.73 57763.1 2221.5 0.4053 95.544 0.531

Reaction catalyzed by [Pd(η^3^-C_3_H_5_)Cl]_2_–**5b** in CH_2_Cl_2_ at room temperature.

# Time Area Height Width Area% Symmetry

1 10.607 249 13.4 0.3091 5.817 0.72

2 14.652 4031.6 183.7 0.3376 94.183 0.764

Reaction catalyzed by [Pd(η^3^-C_3_H_5_)Cl]_2_–**5c** in CH_2_Cl_2_ at room temperature.

# Time Area Height Width Area% Symmetry

1 11.465 2273.3 102.5 0.3242 42.736 0.517

2 16.086 3046 122 0.3816 57.264 0.721

Reaction catalyzed by [Pd(η^3^-C_3_H_5_)Cl]_2_–**4a** in CH_2_Cl_2_ at 0 °C.

# Time Area Height Width Area% Symmetry

1 12.716 301.1 10.9 0.4589 1.893 0.631

2 18.539 15603.6 498.9 0.5213 98.107 0.669
